# History and status quo of higher public health education in China

**DOI:** 10.1186/s40985-020-00120-x

**Published:** 2020-06-01

**Authors:** Hui Jin, Guoqiang Dong, Liling Zou, Xiaobing Shen, Dongmei Li

**Affiliations:** 1 Department of Epidemiology and Health Statistics, School of Public Health, Southeast University, #87, Dingjiaqiao, Nanjing, 21009 China; 2 Department of History, Fudan University, Shanghai, 200433 China; 3 School of Medicine, Tongji University, Shanghai, 200092 China; 4 School of Public Health, Southeast University, Nanjing, 210009 China; 5 Clinical and Translational Science Institute, School of Medicine and Dentistry, University of Rochester, Rochester, NY 14642 USA

**Keywords:** Public Health, Preventive Medicine, Higher Education reform, China

## Abstract

This purpose of the study is to systematically understand the development history and influencing factors of higher public health education in China. We extensively collected the public health education-related literature in China, summarized the general framework, historical origin, and development mode of the existing public health education system, and discussed the potential development tendency. Public health education in China changed with the development of higher medical education and higher education in China. Higher education in China has experienced several large-scale adjustments and enrollment expansions due to different purposes. Therefore, its development stage can be roughly divided into three stages: 1949 to 1976 (period of planned economy), 1977 to late 1998 (period of reform and opening up), and from 1999 to present (period of deepening reform and social transformation). The current public health education in China is influenced by many models, such as the American model, European model (especially the former Soviet Union), and ideological and political education model. It still faces some problems or challenges, such as bachelor’s programs, Master of Public Health, social identity, professional accreditation, and broader public health. In fact, it is necessary to establish an important education system based on the concept of modern public health, beyond the existing medical education system, in order to meet the challenges and needs of public health in the twenty-first century.

## Background

With the rapid development of the global economy, the transformation of human disease and medical models, and the deepening of higher education reform, and new requirements for modern public health education have been forward [1]. How to develop the existing public health model to meet the challenge of public health in the twenty-first century is an important and urgent problem. The study attempts to explain the process of self-improvement by examining the problems and deficiencies in the practice of higher public health education in contemporary China. It is hoped that the study can attract the attention of more scholars and make a more in-depth and systematic analysis of the problems of higher public health in China.

## Basic concept

Hygiene, preventive medicine, and public health are different concepts. Hygiene refers to conditions and practices that help to maintain health and prevent the spread of diseases. It studies the relationship between the external environment factors and human health and expounds the impact of environmental factors on human health. The concept of preventive medicine is more prevalent in China and often replaces the concept of public health. In the 1920s, CEA Winslow [2] wrote: “public health is the science and the art of preventing disease, prolonging life, and promoting physical health and efficiency through organized community efforts …….” According to the definition [3], “preventive medicine is practiced by all physicians to keep their patients healthy. Preventive medicine focuses on the health of individuals, communities, and specific populations. Its goal is to protect, promote, and maintain health and well-being and to prevent disease, disability and death.” Therefore, preventive medicine is a branch of medicine that serves public health, which is at the intersection of medicine and public health [4]. The main difference between preventive medicine and public health is that the former is based on basic medicine and clinical medicine, while the latter is based on social science, behavioral science, economics, statistics, and management. In addition to disease prevention, public health is committed to protecting and promoting community and personal health [5]. Public health education not only covers medical students, but also extends to non-medical students and professional groups [6].

The difference in preventive medicine and public health came from the historical development of public health education in the West. It can be used either as an independent institution (such as the American model) or as part of an academic institution (such as the European model, affiliated to medical schools). The European school of Public Health has been affiliated to the medical profession since its inception, which limits its non-medical development in areas such as sociology and health policy. The rapid development of public health education in the USA benefited from the students of different backgrounds (medical and non-medical). These distinct education models have been integrated into the development of education in China, each of which has had a different impact on the development of China’s higher public health at different stages.

## Historical origin

In the long struggle against nature and diseases, the Chinese people have not only made brilliant achievements in disease treatment, but also accumulated rich ideological knowledge of disease prevention and control, such as “prevention treatment of disease” of *the Yellow Emperor’s Classic of Internal Medicine*. However, this idea has never often been applied to the Chinese people, due to the lack of medical resources, organizational capacity, and the will. Therefore, the theory and educational modes of traditional Chinese medicine were not enough to cope with the challenges of modern public health in the early twentieth century. Based on the social economic and political changes in China and the associated needs, the western public health education system was introduced, and the early public health education was found in China.

The theory and practice of modern public health were developed to cope with the side-effects of the scientific and industrial revolutions. At the beginning of the twentieth century, Chinese public and private universities introduced western education model, mainly providing public health education for medical students. Some examples were as follows: Shanghai German Medical Hall (later Tongji Medical College) in 1907 [7], Peking Union Medical College (later Peking Medical College) in 1912 [8], Private West China Union Medical College (later West China Medical College) in 1914 [9], and National Fourth Zhongshan Medical College (later Shanghai Medical College) in 1927 [10]. These colleges established public health branches/course groups for disease prevention, environmental health, and maternal and child health, as well as public health practice teaching areas for medical students to carry out internships. In 1923, John Grant, a public health professor from Peking Union Medical College [11], founded the first Peking health firm (1925) and Ding county rural health experimental area (1929), which became a model of public health education combined with urban or rural practice (the origin of barefoot doctor) in the world. “This endogenous growth is an exploration of the localization and nationalization of foreign education resources” [12].

However, these explorations were interrupted by social and political factors such as World War II and the Chinese civil war. In contrast, the communist party of China actively explored and formed a new educational concept—“revolution education model” [13]. Its characteristic was that it emphasized the combination of ideological and political education, mass education and productive labor, and practical education, which made a profound impact on higher education reform. In November 1931, the Hygiene School of Chinese workers’ and peasants’ Red Army was founded in Ruijin, Jiangxi province. Under the leadership of the communist party of China, the school trained a leader of military health officials. The school changed its name to Hygiene Department of China Medical University until 1949. It was the first Hygiene Department established in China’s public health education, which marked the beginning of the preventive medicine education system in New China [14].

## Contemporary development

Public health education changed with the development of higher medical education and higher education in China. Its stage can be roughly divided into three stages. The first period was from 1949 to 1976 (the period of planned economy); the second period was from 1977 to the end of 1998 (the period of reform and opening-up); the third period was from 1999 to present (the period of deepening reform and social transformation) [15]. Chinese higher education has experienced several large-scale adjustments and expansions due to different purposes (Table 1) [12]. Table S1 shows more specific events that have taken place in China since 1949. Figures S1 and S2 show the trend based on the school of public health of the two universities.

**Table 1:** the development stages of contemporary higher education and public health education in China [16, 17]

Stage	Time	Characteristics	Purpose of higher education reform	Public health education
First (the formative period)	1949–1956	Expansion and recovery	To meet the needs of national construction and social needs	Imitation of the Soviet model: 1950, the first session on national health; 1953, the 167th meeting of the State Council; 1954, the first national conference on higher medical education
	1957–1966	Expansion	The leap-forward development based on the great leap forward	Deviation from the Soviet model: 1959, national conference for the exchange of teaching experience; 1961, *work regulations about colleges and universities directly under the Ministry of Education (draft)*
	1967–1976	Shrinkage	The extreme political education development	The decade of unrest
Second (the development period)	1977–1985	Expansion	To resume the college entrance examination	Selective recovery and rectification period: 1977, *opinions on the recruitment of colleges and universities and opinions on the recruitment of graduate students in colleges and universities*; 1984, the first academic symposium on professional education in public health; 1985, *the decision of the national central committee on the Reform of Education System*; 1985, the Department of Hygiene was upgraded to school of public health.
	1986–1998	Recovery and adjustment	The connotative development based on structural reform	The establishment of modern university system: 1988, National Conference on higher Medical Education; 1989, the found of Chinese association of public health education of Chinese Preventive Medical Association; 1993, doctoral students of public health were enrolled.
Third (the improvement period)	1999–2005	Expansion	The leap-forward development of college enrollment expansion	The big expansion: 2000, *Reform and Development Compendium of Chinese Medical Education*; 2002, Master of Public Health (MPH) enrollment
	2006	Recovery and adjustment	The connotative development based on the improvement of quality	Meet the needs of health service and improve the quality: 2006, *basic requirements of public health education*; 2009, full-time MPH enrollment; 2009, *National Medium- and Long-Term Program for Education Reform and Development*; 2013, National Conference on Graduate Education Work; 2013, the establishment of the steering committee on public health and preventive medicine teaching in higher education

### The first stage: the formative period (1949–1976)

#### Imitation of the Soviet model from 1949 to 1956

After the social-political system found by the communist party of China in 1949, China’s higher public health education system was initially formed under the background of professionals demand and gradually imitated the Soviet model [18].

### Professional demand

At that time, there were several health problems, such as epidemic infectious diseases, 20% maternal mortality, and prevalence of insect-borne bacteria. In response to these situations, a national policy of “prevention first” was formulated in 1950 and Sanitation Epidemic Prevention Stations (SAPS, now named Center for Disease Prevention and Control, CDC) were established throughout the country in 1953 [19]. The urgent demand for professionals had greatly promoted the rapid development of public health education.

### Establishment of departments

Under the influence of the medical education and health care system of the Soviet Union, some medical colleges in China set up Departments of Hygiene in order to develop public health education according to national policies (Table 2). The educational model of this period was mainly based on the Soviet educational model [20, 21]. At that time, the main characteristics of the model were (1) the organizational structure of “college-department- discipline-teaching team” [22]; (2) the academic year system, no elective courses, and no transfer of majors; (3) subject-centered and unified teaching plans [22]; (4) Soviet textbooks and Russian teaching [22]; (5) the curriculum system included public basic courses (politics, mathematics, physics, chemistry, etc.), basic medical courses (anatomy, physiology.), clinical medicine courses (internal medicine, surgery), professional courses, and internships (clinical practice, professional practice), which constituted the basic framework of preventive medicine for a long time; and (6) the close relationship between Department of Hygiene and SAPS.

**Table 2:** the development of school of public health (preventive medicine) in China from 1949 to 2016

time	unit	number	length^#^
1949	Department of Hygiene of Chinese Medical University	1	
1950	Rename as School of Public Health of Chinese Medical University	1	3
1954	Shanxi Medical College, Shanghai Medical College, Peking Medical College, Shandong Medical College, Sichuan Medical College, Wuhan Medical College, Jiangsu Medical College, Chinese Medical College, and Haerbin Medical College	9	5
1955	Department of Hygiene in Shanxi Medical College, Shanghai Medical College, Peking Medical College, Sichuan Medical College, Wuhan Medical College, Haerbin Medical College	6	5
1958	Six in 1955, plus 17 in Jiangsu, Shandong, Fujian, etc.	23	5
1965	The same as ones in 1955	6	6
1966	Stop enrollment during 1966–1971	6	–
1984	Six in 1955, plus 21 in Anhui Medical College, Fujian Medical College, Guangdong Pharmacy College, Zhongshan Medical College, Guangxi Medical College, Henan Medical College, Hunan Medical College, Baotou Medical College, Nanjing Medical College, Nanjing Railway Medical College, Bethune Medical College, Chinese Medical College, Shenyang Medical College, Ningxia Medical College, Lanzhou Medical College, Tianjin Medical College, Shandong Medical College, Kunming Medical College, Xinjiang Medical College, Wuhan Metallurgy Medical College, and Mudanjiang Medical College	27	5or 6
1985	Department of Hygiene renames as School of Public Health, and Health discipline renamed as preventive medicine discipline	27	5 or 6
1988	Twenty-seven in 1984, plus 5 in Guiyang Medical College, Huabei Coal Medical College, Jiangxi Medical College, Dalian Medical College, and Jining Medical College	32	5
2010	See website	68^*^	5
2016	See website	99^#^	5

*Original from http://www.nseac.com/eva/GEDE.php?DDLThird=211004&DDLyear=2010;

#Original from http://www.nseac.com/html/260/676507.html 2017-1-10

#Original from the history of School of Public Health in Fudan University: http://sph.fudan.edu.cn/a/144

Also, some medical colleges began to enroll postgraduates. Shanghai Medical College enrolled postgraduates in 1956 and 29 graduates in 1966. The Ministry of Health held a series of training courses for leaders and health technicians. For example, from 1953 to 1962, Shanghai Medical College trained more than 400 health management leaders, which was the beginning of a professional diploma (three-year system) for health leaders.

At the early founding of the People’s Republic, Chinese preventive medicine education system had initially been formed, including the foundation of the Department of Hygiene, Secondary Health Schools for formal students, and training organization for health managers and technicians.

### Deviation from the Soviet model from 1957 to 1966

The factors leading to the revolutionary education model of higher public health education were the reflection on the experience and models of the former Soviet Union [13], and the rupture of Sino-soviet relations.

### Sustained development

Teachers began to write their textbooks of preventive medicine instead of using Soviet textbooks. In 1959, the national conference on the exchange of teaching experience and the compilation of teaching materials held in Harbin laid the foundation for the construction of preventive medicine textbook [22]. Teaching contents had been adjusted according to the social needs, such as adding the content about the labor hygiene problems of agriculture, forestry, animal husbandry, and fishery, and the hygiene problems of children participating in production and labor.

### Deviation from the Soviet model

(1) Excessive labor not professional practice was included in the teaching plan. As a result, students had less time to study and were unable to achieve their learning goals. (2) Students were responsible for compiling syllabus and teaching materials, resulting in poor quality of teaching materials [8]. (3) Russian class was replaced by English class in 1960. (4) The discipline of healthcare organization or health management had been canceled. This course mainly introduced the system of health care organizations in the Soviet Union, which was not suitable for China’s actual situation. Due to the breakup of Sino Soviet relations, the course was suspended in 1964, which delayed the discipline for more than 20 years.

### Adjustment of education mode

Due to a series of problems brought about by the previous education revolution, new policies were put forward to “adjust, consolidate, enrich and improve” in 1960 and “Provisional Regulations on institutions of higher education” In 1961, which re-ensured the normal teaching order. The management of higher education had gradually returned to standardization and institutionalization, and the quality of teaching had been further improved. However, productive labor was retained. In 1966, six original health departments were retained, while other additional departments were disbanded.

At this stage, the overall trend of preventive medicine education was in a state of sustainable development. But there were some faults: firstly, under the influence of the “Leftism” trend (pursuing high speed and high indicators) and regardless of objective conditions, the numbers of the Department of Hygiene were blindly expanding. From 1958 to 1962, it expanded from 6 to 23 (Table 2). The number of faculty and staff in some departments was less than 10, coupled with financial difficulties. Secondly, there was a dogmatic tendency in the process of learning from the Soviet Union, that is, copying the teaching contents of the Soviet Union regardless of national conditions, divorced from society, production, and the masses. Thirdly, the existence and development of prevention science cannot be equated with political problems, such as the development of healthcare organization discipline.

### The decade of unrest from 1967 to 1976

During the Cultural Revolution, the extreme performance of “revolutionary education mode” severely damaged the enterprise of public health education [13]. The educational achievements of the past 17 years had been completely denied, and school teachers were dismissed. Public health education had been forced to adjust enrolling three-year students, reducing enrollment numbers, and the numbers of existing teaching plans and textbooks. For example, Department of Hygiene in Shanghai Medical College stopped enrolling students in 1966–1971 and began enrolling three-year students in 1972 (Figure S1).

### The second stage: the development period (1977-1998)

During the time, there happened to be the fundamental shift of the revolution educational model from the class struggling to the talent training, emphasizing that ideological education should not be overriding higher education. This change led to the selective restoration of the Soviet educational model and the introduction of the advanced university system in West. After the Third Plenary Session of the Eleventh Central Committee of the Communist Party of China, SAPS was restored and expanded according to the needs of the people for disease prevention and control. There was an urgent need to supplement various types of health professionals to promote the development of preventive medicine education.

### Selective restoration and rectification period from 1977 to 1985 [23]

From 1977 to 1979, recovery entailed the restoration of the Soviet model science and technology education, and reorganization was an effort to rectify the formal higher education.

### Restoration of the educational system

The recovery of the educational system was reflected in the Department of Hygiene, enrollment, and training organization. Firstly, after 1977, many colleges rebuilt new health departments. The organizational structure had been gradually developed, including health statistics, epidemiology, environmental health, occupational health, nutrition and food health, school health, and general hygiene (for students majoring in non-preventive medicine). In the late 1980s, some medical schools established the Department of Social Medicine and Health Management. Shanghai Medical College opened the major of health management and began to enroll undergraduates in 1985. Secondly, after the resumption of the national college entrance examination, the departments of hygiene of various medical colleges began to resume undergraduate and graduate enrollment. Peking Medical College enrolled 494 students in 1977 and 513 in 1978, and the first batch of graduate students were 126 [22]. Thirdly, in 1981, Harbin Medical College established a health leadership training center and set up a three-year health management cadre major. Followed by Shanghai First Medical College, Wuhan Medical College, Beijing Medical University did. It reflected the recovery from the aspects of increasing professional teachers, improving equipment conditions, revising teaching plans and syllabus, unifying teaching materials, strengthening the construction of teaching base, and so on.

### Preliminary exploration

Although the national preventive medicine teaching plan was issued in 1979 and 1982, respectively, it was no longer mandatory, but provided a reference for the revision of the teaching plan. Educators began to explore how to form a standardized and qualified talent training model in line with their own teaching rules. Under the influence of western education model [24], Chinese scholars discussed three questions during 1978 and 1985: was it necessary to separate preventive medicine education from medical education? Should western public health education replace Chinese preventive medicine? Was it necessary for preventive medicine education to be divided into multiple majors? In 1984, the first academic seminar on health professional education was held in Haerbin city of China (series articles published in No. 6, 1984 of Medical Education (China)). Attendees thought that although schools of public health were affiliated with medical colleges, it should be relatively independent from medical schools and had greater autonomy. The representatives of Peking Medical College proposed the proportion of courses in the teaching plan and the distribution of specialized courses (Table 3) [25]. Haerbin Medical College recommended that the health discipline should be divided into health inspection, public health, epidemiology, and health management [26]. The exploration of these models provided a reference for the next reform of public health education.

**Table 3:** Curriculum of preventive medicine discipline in 1984 [25]

Semester	Course	Note
First	Health organization	To understand the organizational construction of primary healthcare organizations in urban and rural areas.
Second	Introduction of preventive medicine	To understand the general situation, nature and status of preventive medicine, and establish the strategy ideology of preventive medicine.
Third Fourth	The first stage of health statistics and epidemiology	To learn statistical methods and epidemiological research methods to enable students to gradually establish the concept of quality, quantity and population, and the basic ideas and methods based on population research.
Fifth Sixth	Internship	To understand the actual work of professional institutions and provide a basis for perceptual knowledge of follow-up professional courses.
Seventh	Health chemistry, data statistics and analysis, etc.	To continue to strengthen the learning of basic courses in order to provide the linkage between basic courses and professional courses.
Eighth	Specialized courses, for example, the second stage of health statistics and epidemiology	
Ninth	Finished specialized courses	
Tenth	Finished bachelor thesis in the field	To train students to apply the theories, knowledge, and skills they have learned into the field to solve practical problems within a certain range.

At the stage, Chinese public health education included primary health, intermediate health, senior health, professional education, and postgraduation education, which constituted a complete preventive medicine education system. These made a positive contribution to the increase in the number of professionals at all levels [14]. However, the development of public health education was still in the initial stage of exploration. Some essential educational ideas and concepts were unclear, and many positive suggestions had not been popularized, such as introductory courses for freshmen, increasing the number of elective courses and adjusting the curriculum system. Although colleges were allowed to revise their teaching plans, the national teaching plans based on biomedical model were still the primary blueprint.

### The establishment of the modern university system from 1986 to 1998

From the late 1980s to the 1990s, Chinese higher education adopted a steady development policy based on financial pressure. In the process of selective restoration of the higher education model of the Soviet Union, it began to learn from western higher education model and explore the establishment of a modern university system, which became the mainstream of the reform and development of higher education after 1992 [13].

### American model?

Chinese scholars had clarified the similarities and differences between public health and preventive medicine [6], ensuring that Chinese preventive medicine could not be replaced by the public health training model of the USA [21]. Because in China, there was a shortage of doctors (8 per 10,000 persons in China vs 17 per 10,000 persons in the USA) due to the reduction of barefoot doctors, and the model of doctors in the 1980s was different from that of American doctors (Chinese doctors were mainly responsible for the work of specialist hospital, not primary health care). Short-term training or formal education in preventive medicine was needed to meet the shortage of health officials [22]. Considering the difference between preventive medicine and public health, Wan CF suggested a new training model based on western public health education model, after preventive medicine professionals were saturated [5].

### The improvement of teaching quality

A health survey was conducted on the graduates of Peking Medical University from 1953 to 1989. It found [27] students had rich biomedical knowledge, but lacked knowledge of social science, behavioral science, and management; students were also unable to collect and use information, design, implement, and evaluate public health projects. These were the accreditation criteria for public health professionals in the USA since 1946 [6]. Since 1986, some reforms had been carried out. For example, the School of Public Health of Shanghai Medical University had set up a series of teaching departments (Department of health education in 1988, Department of health management, Department of health economics, and Department of hospital management in 1989) to compensate for the lack of knowledge in these areas of education [10]. In order to promote the development of public health practice teaching, many colleges adopted a variety of practice modes [28, 29]. In 1989 and 1990, Tianjin medical school constructed urban and rural practical teaching modes with the combination of main base and satellite bases, which strengthened the operation of practical teaching and the mechanisms of reward and punishment [28]. In 1988, the Chinese Union School of Public Health began to recruit on-the-job postgraduates (the predecessor of the Master of Public Health) to train applied talent in public health [30].

The main problems of this stage were as follows: the introduction of market economy had a great impact on the field of public health, leading to an emphasis on clinical medicine rather than prevention medicine. Attaching importance to scientific research and neglecting teaching research lead to the separation of public health education from public health practice. The education model of the former Soviet Union had become the main factor restricting the development of modern public health education.

### The third Stage: the improvement period (1999-)

#### The “big expansion” from 1999 to 2005

The establishment of the modern university system needs to enhance the social adaptability of the university and meets the needs of people to receive higher education. In 1999, due to the urgent need for a large number of trained professionals, the Chinese government formulated a great strategy to expand the enrollment scale of higher education [16]. In 2000, a total of 2675 students were admitted to the bachelor’s programs in preventive medicine; and by 2010, the number had increased 2.5 times to 6565 [31]. Of historical importance, it made Chinese higher education enter into an internationally recognized popularizing education stage and allowed China to become a great power in higher education [32]. Especially in 2000, many independent medical colleges were merged into universities, so the schools of public health became the parts of the universities [33]. For example, the Peking Medical College was merged into Peking University.

### Training pathway

The increasing number and complexity of public health problems had led to a diversified demand for public health and preventive medicine professionals. For undergraduate education, some colleges and universities adopt the method of “early integration and late differentiation.” In the five-year plan, there were 2.5 years of basic medicine, 1 year of clinical medicine, and 1.5 years of public health. According to the demand of the talent market, the final professional education was divided into preventive medicine, nutritional hygiene, maternal and child health care, health management, and so on. However, in order to avoid over-detailed classification of disciplines, it was adjusted into two majors: preventive medicine and health management.

For graduate education, two talent training models were proposed in 2001, namely, seven-year senior public health management and three-year master’s degree in public health [34]. Subsequently, the former model was conducted in the School of public health of Peking University, delaying the 5 years of preventive medicine to 7 years.The State Council issued “the trial rules on the professional degree of Public Health (Degrees [2001] ninth)”, allowing collegesand universities to recruit masters of public health from 2002 at the government level. Furthermore, full-time MPH enrollment began since 2009.

The connotative development of higher education and the changes of people's disease spectrum put forward new requirements for the reform of public health education. However, there were some problems in undergraduate training, such as the low proportions of practical courses and elective courses, the lack of primary health care content and humanities courses [35], the lack of on-site practical ability, and the lack of practical supervision and evaluation [36, 37]. Although professional postgraduate education in public health has begun to explore, there was indistinguishable between MPH and academic master training model.

### To meet health service needs and improve the quality since 2006

China had carried out a lot of work in the field of higher public health education, and a unique system had been formed in the process of exploring the education model.

### Change of teaching contents [38]

(1) Broad foundation. The proportion of general education courses (humanities education, social sciences, economic management, etc.) and elective courses (freshman seminars, series seminars across the teaching plan) had increased, while the proportion of basic courses had decreased. (2) Interdisciplinary. It had been emphasized about cross-discipline and inter-discipline courses, such as evidence-based medicine, global health, and health technology assessment. (3) Practice and innovation. The student scientific research and training program was first established at Tsinghua University in 1996. At present, it had been widely promoted in Chinese colleges and universities, which had greatly inspired the innovative consciousness and innovative thinking of undergraduates. It was worth noting that the current teaching program had adopted the distribution of specialized courses recommended in 1984 (Table 3), such as the establishment of an introduction to preventive medicine and the transfer of epidemiology and statistics from the fourth year to the sophomore year. The courses provided a good idea for students to contact public health and carry out scientific research. (4) Internationalization. English teaching and bilingual teaching were becoming the mainstream of the education reform. Some methods were adopted, such as introducing classical western textbooks, offering English courses, training teachers abroad, and hiring foreign teachers. For example, these changes had been showed in preventive medicine teaching plan from 1977 to 2017 in school of Public Health, Southeast University (Table S2).

### Core competency

Under the influence of the western education model, Chinese scholars actively explored the definition of the core competencies for public health education in China. On July 23, 2006, the joint meeting of national public health directors approved the basic requirements of public health education, covering 37 items in 6 fields and was recognized by domestic university experts, CDC staff, and students [39, 40] (Table S3). The goal was mentioned to cultivate “public health innovation talents of internationalization” and to carry out comprehensive teaching reform of preventive medicine in the school of public health of Wuhan University [41]. Through literature review, Xiao et al. found that “general course education” should be strengthened to improve students’ knowledge structure, including “management and organization mobilization capacity,” “information collection and analysis capability,” “innovation ability of public health practice research,” and “internationalization of public health education” [39].

### Practical training

The emphasis on practice training originated from the Soviet model; but in 1980s, practical courses were gradually weakened due to economic problems, and then there was a lack of effective supervision and evaluation [42]. In 2009, the government put forward specific requirements for the training of high-level innovative talents [22]. In 2013, it was pointed out that graduate education should actively serve social development [43]. This had promoted the close combination of public health colleges and practical organizations, including cooperative education, two-way teacher exchanges, and the joint establishment of public health research institutes [44]. Supported by Jiangsu CDC and Nanjing Medical College, the first national public health skills competition for college students was held in Nanjing in April 21, 2018. Its aim was to enable students to deal with public health practice issues and to promote the cooperation between universities and CDC.

## Discussions

### Ideological and political education

Both the early and the later target of talent cultivating emphasized the cultivation of professionals who meet the needs of socialism, which reflected the orientation of talent training in the field of ideological education. Over the past 30 years of reform and opening up, the ideological and political education in colleges and universities in China had realized the “transformation”: from the extreme revolutionary model to scientific and reasonable ideological education model, from “taking class struggle as the outline” to “training talents as the center,” and from “society-oriented” to “people-oriented.” It had gradually established the guiding ideology of “people-oriented, establish noble moral values, cultivate people” and established the scientific concept of “education-oriented, moral education first.” These changes had also promoted the development of higher public health education towards a standardized and scientific way.

### Bachelor program

Generally speaking, the history of undergraduate education in preventive medicine was the history of public health education in China. This model, rooted in the hygiene model of the former Soviet Union, integrated into the western public health education model, combined with China’s national conditions, and formed a preventive medicine education system with Chinese characteristics. Based on medical education, it has changed from biomedical model to biological, psychological, and social model. Preventive medicine has been the core major of all public health colleges in China, although it had formed many branches, such as food hygiene and nutrition, maternal and child health, health management, and global health. These branches were also based on medical education and had a similar curriculum structure.

With the change of social demand and the requirements of higher education reform, the curriculum content, curriculum category, and module proportion have been adjusted continuously, such as the curriculum system of Southeast University from 1977 to 2017 (see Table S2). However, it still could not meet to the diversified needs of modern public health, resulting in the lack of experts in health economics, health management, health policies, health-related laws and regulations, and health emergency [1, 45]. This indirectly led to a variety of education models, teaching contents, and curriculum planning (Table S4) adopted by different schools to meet the social needs. Besides, the proportion of medical courses in all courses is still a controversial topic.

Some methods have been tried to adjust and/or integrate the curriculum of preventive medicine, set up a new major (such as Global Health major), extend the length to 7 years of bachelor and master degree (such as MPH), and refer to traditional US mode (4 years in other field + 2–3 years in graduate education of public health). Noticeable, MPH has become the fastest growing degree in China to meet different career needs. Besides, it is positive that continuing education after graduation will be a beneficial complement to public health education.

### MPH

In addition to formal academic master and doctoral degrees, MPH has become the focus of public health education in China in recent years. Undergraduate education, especially preventive medicine, is the starting point of MPH and determines the realization of MPH teaching objectives. Only recruiting-related majors of preventive medicine is not conducive to the development of MPH education. Furthermore, the existing education mode of MPH is rooted on academic master mode, showing a similar trend of “theorization,” which led to the lack of professionalism in curriculum system and training model. Therefore, although the MPH education in China has achieved great-leap-forward development since 2002, the “theorization” training model could not reflect the characteristics of MPH degree itself, and not meet the requirements of social public health for professional degrees.

Some methods are recommended as follows: the prominent feature of MPH is that it is oriented by professional practice and pays attention to the cultivation of students in public health practice. The curriculum system should be guided by the needs of public health profession, and the core goal is to improve students’ core professional competence. It is also necessary to actively promote the docking of MPH degree and professional qualification.

### Social identity

The lack of social identity caused the lower quality and loss of public health workforce. Public health education, derived from preventive medicine, has still been recognized as a part of medicine education, even at the national level [1]. Therefore, compared with clinical medicine, it is difficult to attract high-quality students engaging into public health due to lower remuneration and high intensity. The phenomena were more serious in comprehensive universities than in medical colleges. The proportion of students who transfer from public health major to other majors was up to 30% or 40% in some universities. In addition, some barriers have been set to only enroll public health graduate with similar specialties in China. This led the large gap between supply and demand of public health workforce. For example, there was a shortage of 22 professionals per 100,000 permanent residents in 2013, namely, the total shortfall of 286,000 in China.

Recruiting qualified and capable personnel into the public health field and retaining them in the public health workforce are two important factors. Therefore, the government should give more support to provide time and resources for pursuing recruitment and retention efforts. Recruitment and retention activities will then be informed by evidence of the impact on employment decisions of public health workers.

### Professional accreditation

How does the modern preventive medicine education form a unified and core standard, according to international standards or national standards? Since 1946, the American Council for public health education has gradually established a mature accreditation system [6]. Since the Ministry of Education of China launched the professional accreditation of clinical medicine in 2008, it has promoted positive thinking on the certification of public health education. A joint meeting of national health directors of the Preventive Medicine Teaching Steering Committee of the Ministry of Education of China was held in 2015. It puts forward the basic principle of constructing an educational professional certification system of public health based on China’s current national conditions and international standard. The quality of public health education is closely related to its process. Although the accreditation program was not launched in 2017, it is estimated that the accreditation assessment could be implemented by the third-party agency in the future. Some colleges are also actively seeking international accreditation.

### Broader public health

The development of public health education in China stems from hygiene, through preventive medicine to public health. The definition of public health emphasizes the community concept [2], so the focus of public health education in China should be on the community, namely, community healthcare or basic public health service. While ongoing health reform provides opportunities for community-oriented care, the primary care workers are often employed in different parts of the system rather than focusing on public health. Effective horizontal programs for community care will require multisectoral cooperation between many vertical systems, including family planning, maternal and child health, and chronic diseases. In order to create successful community-based systems, a broader concept of public health is needed, integrating biological, social, and psychological aspects of improving health. This integration also requires high-level support from the Ministry of Health and Ministry of Education to ensure that public health education continues to advance [46].

China’s public health status has benefited from the higher education reform in the last half of the twentieth century. However, the current situation also requires schools of public health achieving recognized international standards to provide the leadership, research, and advocacy necessary to meet the new challenges of public health in rapidly changing societies [47].

## Conclusions

Current public health education in China was influenced by multiple models, including the American model, the European model (especially the former Soviet Union), and the ideological and political education model, and has been gradually formed about the present system of Chinese higher public health education. Indeed, it is necessary to establish an important education system based on the concept of modern public health, beyond the existing medical education system, in order to meet the challenges and demands of public health in the twenty-first century.

## Supplementary information

**Additional file 1: Figure S1**. The enrollment of bachelor in School of Public Health of Fudan University between 1950 and 2000

**Additional file 2: Figure S2**. The enrollment of bachelor and graduate in School of Public Health of Southeast University between 1976 and 2015

**Additional file 3: Table S1**. The important events of contemporary public health education in China. **Table S2**. The changes of the preventive medicine teaching plan from 1977 to 2017 in school of Public Health, Southeast University. **Table S3**. Basic Requirements of Public Health Education in China. **Table S4**. The preventive medicine teaching plan from Chinese universities in 2015

## Data Availability

No data were used to generate this manuscript.

## Abbreviations

CAPHE: Chinese Association of Public Health Education
MPH: Master of Public Health
CDC: Center for Disease Control and Prevention
