# Supplementary figures and images for: History and status quo of higher public health education in China
Source: Public Health Rev. 2020 Jun 1;41:12. doi: 10.1186/s40985-020-00120-x (PMC7262757; doi:10.1186/s40985-020-00120-x)

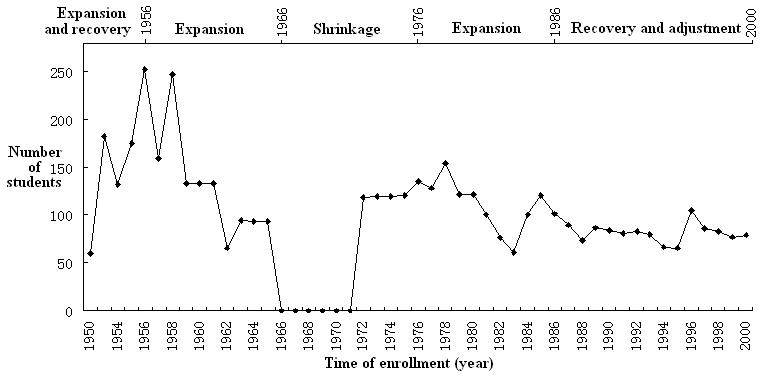

Supplement: Supplementary file 1 — Additional file 1: Figure S1. The enrollment of bachelor in School of Public Health of Fudan University between 1950 and 2000 [file 40985_2020_120_MOESM1_ESM.jpg]

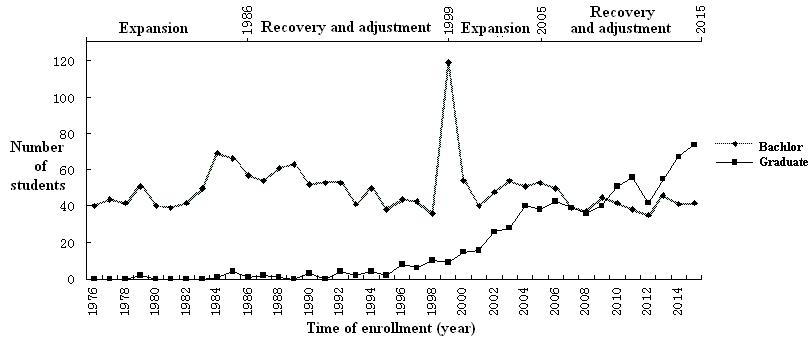

Supplement: Supplementary file 2 — Additional file 2: Figure S2. The enrollment of bachelor and graduate in School of Public Health of Southeast University between 1976 and 2015 [file 40985_2020_120_MOESM2_ESM.jpg]
