# Supplementary material for: History and status quo of higher public health education in China
Source: Public Health Rev. 2020 Jun 1;41:12. doi: 10.1186/s40985-020-00120-x (PMC7262757; doi:10.1186/s40985-020-00120-x)
Supplement: Supplementary file 3 — Additional file 3: Table S1. The important events of contemporary public health education in China. Table S2. The changes of the preventive medicine teaching plan from 1977 to 2017 in school of Public Health, Southeast University. Table S3. Basic Requirements of Public Health Education in China. Table S4. The preventive medicine teaching plan from Chinese universities in 2015 [file 40985_2020_120_MOESM3_ESM.docx]

**Table S1 the important events of contemporary public health education in China**

| Time | Title | Contents |
| --- | --- | --- |
| 1950 | The first session on national health | To established a policy of prevention first |
| 1951 | *Decision on the Development of Health Education and the Training of Health Workers at All Levels* |  |
| 1953 | The 167th meeting of the government of the state council | To approve the establishment of national sanitation epidemic prevention stations |
| 1953 | The fourth meeting of First session of the Chinese People's Political Consultative Conference | Mao Zedong proposed "learn from the Soviet Union". Medical colleges carry out educational reform. |
| 1953 | State Council informed the adjustment of schools of Colleges |  |
| 1954 | The first National Conference on higher medical education | The length of hygiene specialty has been changed from 4 years to 5 years. A unified teaching plan has been formulated and the principles for compiling the syllabus have been stipulated. |
| 1955 | The Ministry of Health determines the adjustment plan of departments of hygiene |  |
| 1957 | *On the Correct Handling of the Contradictions among the People* | Mao Zedong pointed out that our educational policy should enable the educatees to develop in moral education, intellectual education and sports and become educated workers with socialist consciousness. |
| 1958 | *Directive on Education Works* | The Central Committee of the Communist Party of China and the State Council put forward the educational policy of combining education with productive labor and serving proletarian politics. The production labor is officially included in the teaching plan. Teachers and students take part in smelting iron and steel, running factories, eliminating diseases and eliminating diseases in the countryside. |
| 1959 | National Symposium on medical education | The view and estimate of the great development of medical education and the Great Revolution in 1958 were reviewed to examine the existing problems in the current work of higher medical education, and how to further consolidate and improve the quality. |
| 1959 | A national conference on teaching experience exchange and textbook compilation | The meeting discussed the implementation of the Party's educational policy and the combination of theory with practice; a collaboration draft between department of hygiene and sanitation epidemic prevention stations; the foundation for the construction of preventive medicine textbook |
| 1961 | *Several Interim Provisions on Teaching in Higher Medical Colleges* | It was necessary to strengthen the basic theory study, basic technical training, experimental operation and necessary systematic professional knowledge teaching. The educational system was basically unchanged. Labor time should not be excessive. Pay attention to work and rest. |
| 1961 | *Several Opinions on Revising the Teaching Plan of Medical Colleges and Universities (Draft)* | The Ministry of Health issued put forward the total number of school hours for each major; political lessons and political activities accounted for 10% of the total school hours; it was not advisable to arrange too much labor. The curriculum was relatively stable. |
| 1961 | *Work regulations about colleges and universities directly under Department of Education (Draft)* | 60 items of Higher Education. |
| 1961 | National Conference on higher medical education | It discussed and implemented the 60 items of higher education, summarized the experience of higher medical education in the three periods since the founding of the People's Republic of China, calls for consolidating achievements, overcoming shortcomings, resolutely implementing the eight-character policy of "rectifying, consolidating, enriching and improving" of the Central Committee and improving the quality of education. |
| 1961 | The second National Conference on adjustment of institutions of higher learning and secondary schools | Conference studies further reduced the scale of education and solved the problems of colleges and secondary vocational schools. Most of the newly established medical colleges and departments had been abolished. |
| 1962 | *The Notice on Changing the Educational System of Medical Colleges and Universities* | The educational system of hygiene specialty had been changed from five years to six years. |
| 1964 | National Conference on medical education | The Department of Medical Education investigated the teaching quality of Harbin Medical College, Jilin Medical College, Shenyang Medical College and Dalian Medical College for 2 months. |
| 1965 | National Conference on higher medical education | It was clear that higher medical education must face the countryside, go out of school, set up teaching bases in the countryside, adhere to the policy of "walking on two legs", coexist long-term and short-term schooling, and give priority to short-term schooling. The conference proposed that the medical profession should be changed from six years to five years. |
| 1967 | *Chairman Mao Zedong's Theory on the education revolution* | It was pointed out that education must serve the proletariat politics and must be combined with productive labor. The working people should be knowledgeable and intellectuals should be laboring. |
| 1970 | The enrollment of worker-peasant-soldier students | Approved by the State Council, Beijing Medical College recruited three year worker-peasant-soldier students. |
| 1973 | Exchange meeting for health professional education revolution experience | The meeting discussed whether there should be the existence of the Department of Health and ensured to develop it, unified thinking and understanding, to promote the development of health profession. |
| 1977 | *Opinions on the Recruitment of Colleges and Universities in 1977* and *Opinions on the Recruitment of Graduate Students in Colleges and Universities* | It became an important turning point for the development of higher education in China. Medical specialties were formulated for five years. |
| 1978 | Plans for publishing textbooks | The Ministry of health compiled plans for publishing textbooks for medical specialties. |
| 1978 | Unified teaching plans | The Ministry of Health issued a trial draft of seven major teaching plans for medical colleges and universities, which began in the autumn of 1978. |
| 1978 | The enrollment of graduate students | 11 colleges and universities directly under the Ministry of health, including Beijing Medical College, resumed the enrollment of graduate students. |
| 1978 | Medical education planning conference | To strengthen the teaching of basic theory, holding postgraduate classes, piloting medical foreign language classes and improving teaching conditions. |
| 1978 | Restore the enrollment system of colleges and universities. |  |
| 1980 | National Conference on higher medical education | The central task of higher medical education was to shift the focus of work to improving the quality of education and train qualified senior medical and health personnel. |
| 1981 | Higher health management enrollment | The Health Cadre Training Center of Harbin Medical College held the specialty of higher health management and enrolls 30 students in three provinces of northeast China. |
| 1981 | Working conference of medical colleges and Universities | To strengthen the ideological and political education of students and the Five-fixedness (assignment, specialty, school system, scale, numbers of students and teachers). |
| 1982 | the found of Health leader’s training center | The Ministry of Health decided to set up a training center for health leaders in seven colleges, including Harbin Medical College, Beijing Medical College, Beijing College of Traditional Chinese Medicine, Sichuan Medical College, Wuhan Medical College, Xi'an Medical College and Shanghai First Medical College. |
| 1982 | Three years of specializing in health management leaders | The Ministry of Health asked Harbin Medical College, Wuhan Medical College and Shanghai first medical college to hold three years of specializing in health management leaders. |
| 1982 | Unified teaching plans | Five year teaching plan for oral medicine, hygiene, paediatrics and four year pharmacy in medical colleges and Universities. |
| 1983 | The establishment of National Association of medical education of Chinese Medical Association |  |
| 1983 | Conference on compilation of teaching materials for health professions |  |
| 1983 | *Notice on setting up elective courses or lectures on health economics in medical colleges and Universities* |  |
| 1983 | The first National Conference on education with electrical audiovisual aids |  |
| 1984 | *Opinions on setting up the course of literature retrieval and utilization in Colleges and Universities* |  |
| 1984 | The first health professional education academic seminar |  |
| 1984 | *The Notice on the Pilot Running of Graduate Schools in 22 universities* | To include Beijing Medical College and Shanghai first medical college. |
| 1985 | *The Decision of the National Central Committee on the Reform of Education System* | It was clearly pointed out that it should reduce essential courses, increase elective courses, and implement credit system and double degree system. It marked the period of comprehensive reform of China's higher education. |
| 1985 | Name changes | The medical schools have been upgraded to universities since 1985, and the departments of Hygiene were upgraded to schools of public health at the same time. |
| 1986 | *Notice on Further Strengthening Ideological Education for college graduates* | The Notice analyses and studies the ideological characteristics of graduates in the new period, and calls for the mobilization of various personnel to do a good job in ideological education of graduates. |
| 1986 | *Notice on the Reform of Ideological and Political Theory Courses in Colleges by the Central Committee of the Communist Party of China* |  |
| 1988 | National Conference on higher medical education | To strengthen preventive medicine education and teaching reform, students' ideological and political work, and to develop continuing medical education and adult education. |
| 1988 | On-the-job graduate students enrollment | Chinese academy of preventive medicine, Peking union medical college, and Peking medical university set up the Chinese Union School of Public Health, which began to recruit on-the-job graduate students and worked to cultivate applied talent in public health. |
| 1989 | The found of Chinese Association of Public Health Education (CAPHE) of Chinese Preventive Medical Association |  |
| 1993 | The enrollment of doctor of philosophy of public health and preventive medicine |  |
| 2000 | The combination between independent medical colleges and universities |  |
| 2000 | *Chinese Medical Education Reform and Development Compendium* | Ministry of Health and Ministry of Education jointly issued and aimed to adjust "Priority development of secondary medicine education" in the early years to "expand higher medical education and reduce secondary medical education". |
| 2002 | Master of Public Health (MPH) enrollment |  |
| 2006 | *The Public Health Education Basic Requirements* | *The Public Health Education Basic Requirements* were approved by the Joint Session of the National Public Health Director. |
| 2009 | Full-time MPH enrollment |  |
| 2009 | *National Medium- and Long-term Program for Education Reform and Development* |  |
| 2013 | National Graduate Education Work Conference | The state explicitly pointed out that the development of postgraduate education in our country should take "the service demand, improve the quality" as the main line, and that the national graduate students education should actively serve the social development. |
| 2013 | The found of Public Health and Preventive Medicine Teaching Steering Committee of Colleges and Universities | In order to improve the quality of talent training, the Ministry of Education hired experts to establish Public Health and Preventive Medicine Teaching Steering Committee of Colleges and Universities, which held public health education meeting regularly with CAPHE, Chinese Preventive Medicine Education Research Association (established in 2005) , and Deans of School of Public Health. |

**Table S2. The changes of the preventive medicine teaching plan from 1977 to 2017 in school of Public Health, Southeast University**

| Year | Total credits | Course (%) | | | | Core module (%) ^#^ | | | | Class Hours^##^ | | | | Practice courses (weeks) | | |
| --- | --- | --- | --- | --- | --- | --- | --- | --- | --- | --- | --- | --- | --- | --- | --- | --- |
|  |  | Required | Limited  elective | Freely  elective | Practice | General  education | Basic Medicine | Clinical Medicine | Preventive Medicine | Total | Theory | Experiment | Optional | Clinical | Professional | Graduation thesis |
| 1977 | - | 100.0 | - | - | NA | 28 | 32 | 24.3 | 15.7 | 3838 | 1954 | 1884 | - | 16 | 20 | - |
| 1978 | - | 100.0 | - | - | NA | 30.4 | 30.9 | 23.6 | 15.1 | 3990 | 1958 | 2032 | - | 16 | 20 | - |
| 1982 | - | 100.0 | - | - | NA | 26.8 | 29.1 | 23.2 | 20.9 | 3964 | NA | NA | NA | 16 | 8 | 12 |
| 1986 | - | 96.8 | 3.2 | | NA | 31.3 | 27.2 | 22.2 | 19.3 | 4055 | 2334 | 1721 | 130 | 16 | 8 | 12 |
| 1991 | - | 91.1 | 8.9 | | NA | 28.1 | 30.0 | 20.9 | 21.1 | 4053 | 2609 | 1444 | 360 | 16 | 8 | 12 |
| 1995 | 174 | 63.0 | 27.0 | 10.0 | NA | 30.8 | 28.0 | 21.4 | 19.8 | 3811 | 2319 | 1492 | 270 | 16 | 8 | 12 |
| 1997 | 219 | 52.3 | 23.1 | 6.8 | 17.8 | 32.6 | 28.9 | 18.9 | 19.6 | 3918 | 2117 | 1531 | 270 | 19 | 8 | 12 |
| 1999 | 225 | 52.2 | 23.8 | 6.7 | 17.3 | 33.0 | 28.0 | 18.4 | 20.6 | 4062 | 2226 | 1566 | 270 | 17 | 6 | 11 |
| 2002 | 292 | 54.3 | 26.9 | 5.1 | 13.7 | 34.1 | 18.5 | 27.0 | 20.4 | 4118 | 2640 | 1208 | 270 | 17 | 6 | 11 |
| 2003 | 253 | 61.7 | 15.6 | 4.7 | 18.0 | 29.3 | 27.1 | 18.8 | 24.8 | 3896 | 2546 | 1158 | 192 | 17 | 6 | 12 |
| 2005 | 251 | 52.0 | 24.3 | 5.6 | 18.1 | 27.6 | 27.8 | 18.8 | 25.8 | 3954 | 2538 | 1128 | 288 | 17 | 6 | 12 |
| 2007 | 200 | 59.2 | 18.8 | 7.0 | 15.0 | 29.7 | 26.6 | 13.7 | 30.0 | 3384 | 2080 | 988 | 316 | 18 | 10 | 8 |
| 2009 | 200 | 51.8 | 16.8 | 15.5 | 15.9 | 31.0 | 25.8 | 15.4 | 27.8 | 3310 | 1784 | 948 | 580 | 18 | 10 | 8 |
| 2011 | 190 | 53.4 | 8.7 | 20.0 | 17.9 | 36.3 | 21.4 | 12.0 | 30.3 | 3444 | 1712 | 900 | 832 | 18 | 10 | 8 |
| 2013 | 190 | 50.3 | 12.6 | 20.0 | 17.1 | 35.9 | 19.9 | 12.6 | 31.6 | 3460 | 1724 | 884 | 852 | 12 | 12 | 18 |
| 2015 | 190 | 49.2 | 15.5 | 17.4 | 17.9 | 33.5 | 22.5 | 10.5 | 33.5 | 3280 | 1752 | 840 | 688 | 12 | 12 | 18 |
| 2017 | 190 | 49.2 | 15.5 | 17.4 | 17.9 | 33.5 | 22.5 | 10.5 | 33.5 | 3280 | 1752 | 840 | 688 | 12 | 12 | 18 |

# excluded freely elective course and practice course. ## excluded practice course. In 1980, “mathematics” was added. In 1982, “mathematics” was renamed as “higher mathematics” and “social medicine” was added. In 1991, “social medicine” was renamed as “health management and social medicine” and “legal foundation” was added. In 1999, “health education” was added. In 2002, literature retrieval course was added. In 2003, the 2 credits of “social practice” were defined as 2 credits for extracurricular research (including student research training project, SRTP). In 2011, introduction on freshmen and seminar courses were added. In 2013, clinical practice had been shortened and professional practice and graduation thesis had been extended; basic public health training courses were added into the third semester in order to help students understand the basic work of scientific research; some courses, “global health”, “emergency public health”, “evidence-based medicine” were added.

Table S3 **Basic Requirements of Public Health Education in China***

| Domain | Content |
| --- | --- |
| Domain 1 | Professional Spirit |
|  | To consciously establish, strengthen and maintain the professional value of public health.  1. To understand the basic ethical norms, ethical principles and legal responsibilities of public health profession, and the role of public health in human survival and social development.  2. To fulfill the lofty mission of safeguarding and promoting health with a rigorous scientific attitude, a high degree of professionalism and a strong sense of social responsibility.  3. To cherish humanity with deep humanistic care and fear life.  4. To safeguard the fairness of health services and safeguard public health interests.  5. To respect cultural diversity, understand public health related sub culture, respect personal rights and privacy.  6. To respect intellectual property rights and abide by academic ethics.  7. To have the sense of autonomy and lifelong learning to adapt to the rapid changes in technology and society.  8. To have positive cooperation attitude, good team spirit and social work adaptability. |
| Domain 2 | Medical Basis  To learn and use the basic medical knowledge and skills correctly.  9. To understand the structure and function of normal human body and understand the physiological and biochemical mechanisms of maintaining body balance.  10. To master the role of genetic and environmental factors in the body and its mechanism.  11. To understand the physiological, psychological and behavioral characteristics of human life cycle and its impact on health.  12. To master the abnormal changes of body structure and function in disease status.  13. To be familiar with the principles of diagnosis and treatment of common diseases.  14. To have the ability of clinical identification of diseases and life-threatening emergencies with greater public health significance, and to master their basic principles of treatment. |
| Domain 3 | Public health |
|  | To firmly establish the population concept, deeply understand the ecological health mode, and apply relevant knowledge and skills.  15. To master the skills of investigating, monitoring the distribution of disease and public health events in the population and their influencing factors, and has the basic ability to formulate intervention strategies and evaluate the effectiveness of intervention.  16. To understand the relationship between natural and social environment factors, genetic and psychological behavioral factors and group health.  17. To understand the health problems and health care needs of women and children, adolescents, the elderly and the disabled, as well as the occupational population.  18. To have the basic skills in field sampling and rapid detection of biological and physical and chemical factors, as well as hygienic and safety assessment.  19. To be awareness of actions to be taken to prevent disease and injury and to promote the health of individuals, families and communities.  20. To have the ability to diagnose community public health problems, propose health promotion strategies, carry out health education and disease prevention services, and develop basic skills for health risk assessment and control.  21. To have the basic knowledge and principles for identifying and warning all kinds of public health emergencies and crises. |
| Domain 4 | Management and social mobilization |
|  | To have the awareness of modern management concepts, related knowledge and skills, and mobilization of health related resources.  22. To understand the various elements and operational mechanisms of the health system, especially the law enforcement departments of disease prevention and control and health supervision, as well as the basic principles of public health service management.  23. To understand and analyze the basic knowledge of health resource allocation, equity and efficiency of health services.  24. To have the basic knowledge and skills of designing, implementing and evaluating public health projects.  25. To have the awareness of health policy development and understand the basic knowledge of health policy analysis and assessment.  26. To have the basic knowledge and skills of evidence-based thinking and evidence-based management and decision-making.  27. To be familiar with health-related laws and regulations, technical specifications and standards, with the basic ability to implement health supervision, monitoring and disease control according to law.  28. To have the basic skills for effective oral and written communication and interaction with government departments, relevant agencies and organizations, the media, the public, colleagues and other health professionals.  29. To have the basic ability to promote the awareness of the government and related departments to deal with public health issues, and to plan and mobilize health-related resources from a professional perspective.  30. To understand the state and dynamics of global public health and the roles of various international health organizations and relevant non-governmental organizations. |
| Domain 5 | Information Management |
|  | To correctly collect and analyze all kinds of health related information and use them reasonably in practice.  31. To have the ability to qualitatively investigate, and the ability to collate, summarize, and refine qualitative data.  32. To have the ability to collect, analyze, interpret and express quantitative data.  33. To use modern information technology to retrieve and analyze health related information from various data sources.  34. To have the ability to compare and judge various types of information from different sources, discover problems from them, and make effective use of information in analyzing or solving problems. |
| Domain 6 | Scientific Research |
|  | To critically evaluate existing knowledge, technology and information, and conduct scientific research in professional activities.  35. To maintain professional sensitivity and explore the curiosity of unknown or uncertain things.  36. To master the basic ability of scientific research thinking, put forward research questions and conduct scientific research.  37. To have the ability to summarize the literature and report the findings. |

*On July 23th, 2006, the public health education basic requirements were approved by the Joint Session of the National Public Health Director.

**Table S4. The preventive medicine teaching plan from Chinese universities in 2015***

| University | Core module (%) | | | | | Practice courses (weeks) | | | | Total credits |
| --- | --- | --- | --- | --- | --- | --- | --- | --- | --- | --- |
|  | General education | Basic Medicine | Clinical Medicine | Preventive Medicine | Total(credits) | Clinical | Professional | Graduation thesis | Total |  |
| Huazhong Technology University | 30.34 | 28.28 | 23.45 | 17.93 | 217.5 | 24 | 14 | 4 | 42 | 292.5 |
| Zhengjiang University | 41.47 | 22.94 | 12.06 | 23.53 | 170 | 18 | 18 | 16 | 52 | 205 |
| Sichuan University | 26.67 | 34.24 | 10.3 | 28.79 | 165 | 20 | 12 | 20 | 52 | 210 |
| Zhongshan University | 35.18 | 25.63 | 19.35 | 19.85 | 199 | 12 | 16 | 18 | 46 | 255 |
| Zhongnan University | 45.82 | 15.48 | 17.34 | 21.36 | 161.5 | 20 | 35 | | 55 | 257.5 |
| Zhengzhou University | 37.14 | 26.86 | 15.43 | 20.57 | 175 | 24 | 17 | | 41 | 245 |
| Tianjin Medical College | 35.22 | 34.22 | 9.97 | 20.6 | 150.5 | 18 | 10 | 25 | 53 | 220.5 |
| Capital Medical College | 34.28 | 22.16 | 20.62 | 22.94 | 194 | 12 | 12 | 24 | 48 | 253 |
| Xiamen University | 25.15 | 40.72 | 14.97 | 19.16 | 167 | 16 | 16 | | 32 | 218 |

*Lu Jie, Wang Yiran, Zhang Xiaofeng, Gao Pan, Lv Quanjun. Study on training programs for preventive medicine undergraduates in China's colleges and universities. Chin J Med Edu Res, 2017, 16(11):1097-1102.
